# Comparative Study of Functional and Radiological Outcomes of Antegrade Versus Retrograde Titanium Elastic Nailing for Pediatric Ulnar Diaphyseal Fractures

**DOI:** 10.7759/cureus.107334

**Published:** 2026-04-19

**Authors:** Ankit Hooda, Hariprasad Seenappa, Tarun Kumar Somisetty Venkata Sai

**Affiliations:** 1 Orthopedics, Sri Devaraj Urs Medical College, Kolar, IND; 2 Orthopedics, R L Jalappa Hospital &amp; Research Centre, Kolar, IND

**Keywords:** antegrade nailing, elastic stable intramedullary nailing, pediatric forearm fracture, retrograde nailing, titanium elastic nail, ulna fracture

## Abstract

Background

Pediatric forearm fractures account for approximately 40% of childhood skeletal injuries, with ulnar fractures being particularly prevalent. Titanium Elastic Nailing (TEN) offers an effective minimally invasive option for these injuries; however, the optimal nail entry point - antegrade (olecranon) versus retrograde (distal ulna) - remains debated. This study compared functional and radiological outcomes, complication profiles, and implant-related morbidity between the two approaches.

Methods

A retrospective comparative study was conducted from May 2023 to May 2025 at the R.L. Jalappa Hospital and Research Centre, Karnataka, India. Seventy children aged 5-16 years with closed ulnar diaphyseal fractures treated with TEN were divided into antegrade (n=35) and retrograde (n=35) groups. Radiographic union time and six-month Disabilities of the Arm, Shoulder and Hand (DASH) scores were the primary outcomes. Bonferroni correction was applied to control for comparisons of multiple complications.

Results

Retrograde nailing showed faster unadjusted union (7.4 ± 0.6 vs. 8.6 ± 0.7 weeks; p<0.001), but the retrograde group had more distal-third fractures. After adjusting for fracture location, the difference narrowed to 0.6 weeks - statistically significant but unlikely to change clinical practice. Functional outcomes were equivalent at six months: full range of motion (ROM) in 91.4% vs. 94.3%, mean DASH scores of 4.2 vs. 3.6 (p=0.198), and near-normal elbow and forearm motion in both groups. The overall complication-free rate strongly favored retrograde nailing (85.7% vs. 42.9%; p<0.001). Olecranon irritation (34.3%) and early implant removal (22.9%) were exclusive to the antegrade group. Two young retrograde patients had nail entry close to the distal physis. Neither showed growth arrest at six months, though longer follow-up is needed.

Conclusion

Both techniques achieve excellent functional outcomes. The advantage of union time in the case of retrograde nailing was attenuated after fracture-location adjustment. The clearest difference was a significantly lower overall complication burden with retrograde nailing (p<0.001), supporting its preferential use for midshaft and distal fractures in older children. However, careful consideration is required regarding potential physeal damage.

## Introduction

Pediatric forearm fractures are among the most common skeletal injuries in childhood, comprising approximately 40% of all pediatric fractures [1]. Ulnar diaphyseal fractures - occurring either in isolation or as part of both-bone forearm injuries - peak in incidence between five and 14 years of age and show a consistent male predominance [2]. Forearm bones in children are more pliable and heal faster than in adults, but high-energy falls and sports injuries frequently produce unstable patterns that cannot be managed conservatively [3]. The Titanium Elastic Nailing System (TENS), or Elastic Stable Intramedullary Nailing (ESIN), has become the surgical standard for pediatric forearm shaft fractures. Its advantages over conventional open reduction and internal fixation (ORIF) with plates include minimal soft tissue dissection, preservation of periosteal blood supply, shorter operative time, and earlier mobilization, all translating to faster functional recovery [4]. Despite the widespread adoption of TENS, the optimal entry point for ulnar nailing remains contested. Antegrade nailing via the olecranon is mechanically reliable and technically intuitive, particularly for proximal-third fractures [5]. The trade-off, however, is a recognized risk of olecranon bursitis, painful hardware prominence, and soft tissue irritation that frequently necessitates early implant removal [6]. Retrograde nailing through a distal ulnar entry avoids these olecranon-related complications and is generally preferred for midshaft and distal fractures but raises concern about inadvertent physeal injury, especially in younger children with a widely open distal ulnar physis, potentially resulting in growth arrest or angular deformity [7]. Existing literature is inconclusive. Johnson et al. reported an increased incidence of problematic implants with antegrade methods, primarily attributed to irritation at the olecranon entry site [6], while Thürig et al. endorsed retrograde nailing for specific fracture patterns, noting few soft tissue problems when performed correctly [8]. Few studies have systematically compared both techniques using validated functional outcome measures. Most surgeons choose an approach based on habit and training rather than data. This retrospective study was designed to address this gap. The primary aim was to compare radiographic union time and six-month disabilities of the arm, shoulder and hand (DASH) score between antegrade and retrograde TEN in pediatric closed ulnar diaphyseal fractures. Secondary aims were to compare range of motion (ROM) recovery and implant-related complication profiles, including the rate of early hardware removal, between the two techniques.

## Materials and methods

Study design and reporting

This retrospective comparative study was conducted at the R L Jalappa Hospital and Research Centre, Kolar, Karnataka, India, between May 2023 and May 2025. Reporting followed the Strengthening the Reporting of Observational Studies in Epidemiology (STROBE) guidelines [9] for observational studies. Institutional Ethics Committee approval was obtained (SDUAHER/R&D/CEC/SDUMC-PG/278/NF/-2025-26) in accordance with the Declaration of Helsinki. Due to the retrospective design, informed consent was waived.

Study population and sampling

A total of 70 pediatric patients were enrolled in this study, comprising 35 patients in the antegrade TENS group and 35 patients in the retrograde TENS group. Sample size was calculated using the formula for comparison of two proportions based on implant complication data (antegrade: 46.8%; retrograde: 18.2%) from Johnson et al. [6]:

n = (Zα/2 + Zβ)² × 2P(1 − P) / (P₁ − P₂)²

Where Zα/2 = 1.96 (two-sided significance level of 0.05), Zβ = 0.84 (80% power), P1 = 0.468, P2 = 0.182, and P (average proportion) = 0.325. The calculated sample size required was 32 patients per group; therefore, 35 patients per group were enrolled to account for potential data loss.

Participants

Children aged five to 16 years with closed ulnar diaphyseal fractures treated using TENS and a minimum six-month follow-up were included. Exclusion criteria comprised open fractures, pathological bone disease, previous surgery on the affected limb, and incomplete records.

Surgical technique

All procedures were performed under general anesthesia with fluoroscopic guidance by single senior orthopedic surgeon. Nail diameter was selected as approximately 80% of the narrowest medullary canal diameter. Each nail was pre-bent to approximately 30° with the apex positioned at the fracture site to generate the corrective force necessary for reduction maintenance [10].

Antegrade Approach

A 1-2 cm longitudinal incision was made at the tip of the olecranon with a triceps split. The cortex was breached using a bone awl directed at an angle of approximately 45° to the shaft axis, and the nail was advanced distally under fluoroscopic control across the fracture site. The proximal end was bent at 90°, trimmed, and left protruding 5-10 mm subcutaneously to facilitate subsequent removal while minimizing soft tissue irritation [10].

Retrograde Approach

A 1-2 cm incision was placed over the dorso-ulnar aspect of the distal forearm, centered approximately 2 cm proximal to the distal ulnar physis. The internervous interval between the flexor carpi ulnaris and the extensor carpi ulnaris was developed by blunt dissection, with protection of the dorsal sensory branch of the ulnar nerve throughout. The entry point was confirmed fluoroscopically, at least 2 cm proximal to the physis. The entry portal was created with a bone awl directed proximally, and the nail was advanced in retrograde fashion across the fracture under fluoroscopic guidance. The distal tail was left protruding 5-10 mm [11].

Postoperative protocol

A volar below-elbow splint was applied for two weeks, followed by supervised mobilization. Follow-up occurred at six weeks, three months, and six months with clinical and radiographic assessment at each visit.

Outcome Measures

Radiographic union was defined as bridging callus across at least three cortices on anteroposterior and lateral radiographs. Functional outcome was assessed at six months using the DASH questionnaire, a validated 30-item instrument with scores ranging from 0 (no disability) to 100 (maximal disability) [12]. ROM was measured goniometrically and included elbow flexion, elbow extension deficit, and forearm pronation and supination.

Statistical Analysis

Continuous variables were analyzed using independent t-tests or Mann-Whitney U tests based on normality assessed by the Shapiro-Wilk test. Categorical variables were compared using the chi-squared test or Fisher's exact test. Analysis of covariance (ANCOVA) adjusted for fracture location. Bonferroni correction (p<0.0083) controlled for multiple comparisons. Statistical significance was set at p<0.05. All analyses were performed using the IBM SPSS Statistics for Windows, Version 26 (Released 2019; IBM Corp., Armonk, New York, United States).

## Results

Demographic and baseline characteristics

Seventy patients met the inclusion criteria during the study period and were included in the final analysis. There were 35 patients in the antegrade group and 35 patients in the retrograde group. Demographic and baseline characteristics were comparable between groups (Table 1).

**Table 1:** Demographic and baseline characteristics of the study population Values are presented as mean ± SD or n (%). p<0.05 considered statistically significant. Continuous variables compared using independent t-test (t-value and df reported); categorical variables compared using chi-squared test (χ² and df reported); fracture location compared using chi-squared test (df=2). For fracture location subgroups, test statistic and p-value are reported on the header row. SD: standard deviation; df: degrees of freedom.

Characteristic	Antegrade (n=35)	Retrograde (n=35)	Test Statistic	p-Value
Mean age (years)	9.8 ± 2.4	10.1 ± 2.6	t = 0.50; df = 68	0.621
Male-to-Female subject ratio	22:13	20:15	χ² = 0.24; df = 1	0.624
Right : Left	19:16	17:18	χ² = 0.16; df = 1	0.631
Fracture location				
Proximal third	8 (22.9%)	5 (14.3%)	χ² = 2.09; df = 2	0.352
Middle third	18 (51.4%)	16 (45.7%)		
Distal third	9 (25.7%)	14 (40.0%)		
Mean time to surgery (days)	2.1 ± 0.9	2.3 ± 1.0	t = 0.93; df = 68	0.389
Associated radius fracture	12 (34.3%)	14 (40.0%)	χ² = 0.25; df = 1	0.619

The mean age was 9.8 ± 2.4 years in the antegrade group and 10.1 ± 2.6 years in the retrograde group (t=0.50; df=68; p=0.621). Midshaft fractures predominated in both groups. Associated radius fractures were present in 12 (34.3%) patients in the antegrade group and 14 (40.0%) patients in the retrograde group (p=0.619).

Radiographic and functional outcomes

**Figure 1:**
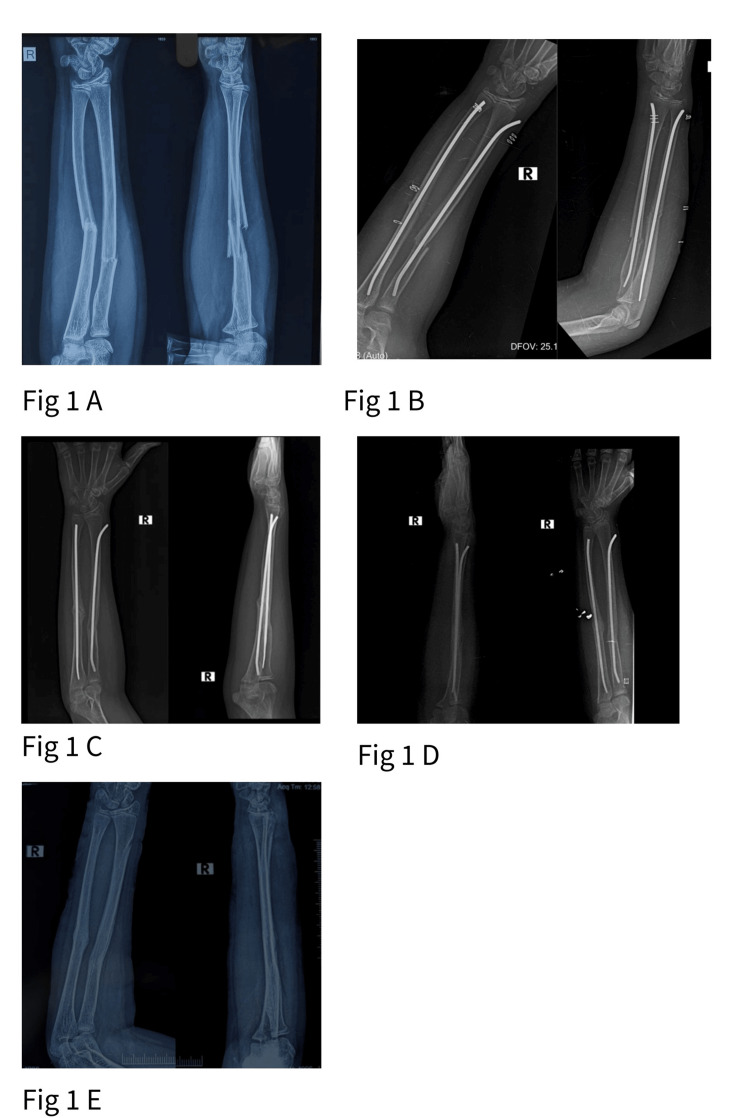
Plain radiographs representing retrograde titanium elastic nailing for right ulna fracture A: Pre-operative; B: Immediate post-operative; C: 6 months follow-up; D: 9 months follow-up with complete union; E: post-implant removal.

**Figure 2:**
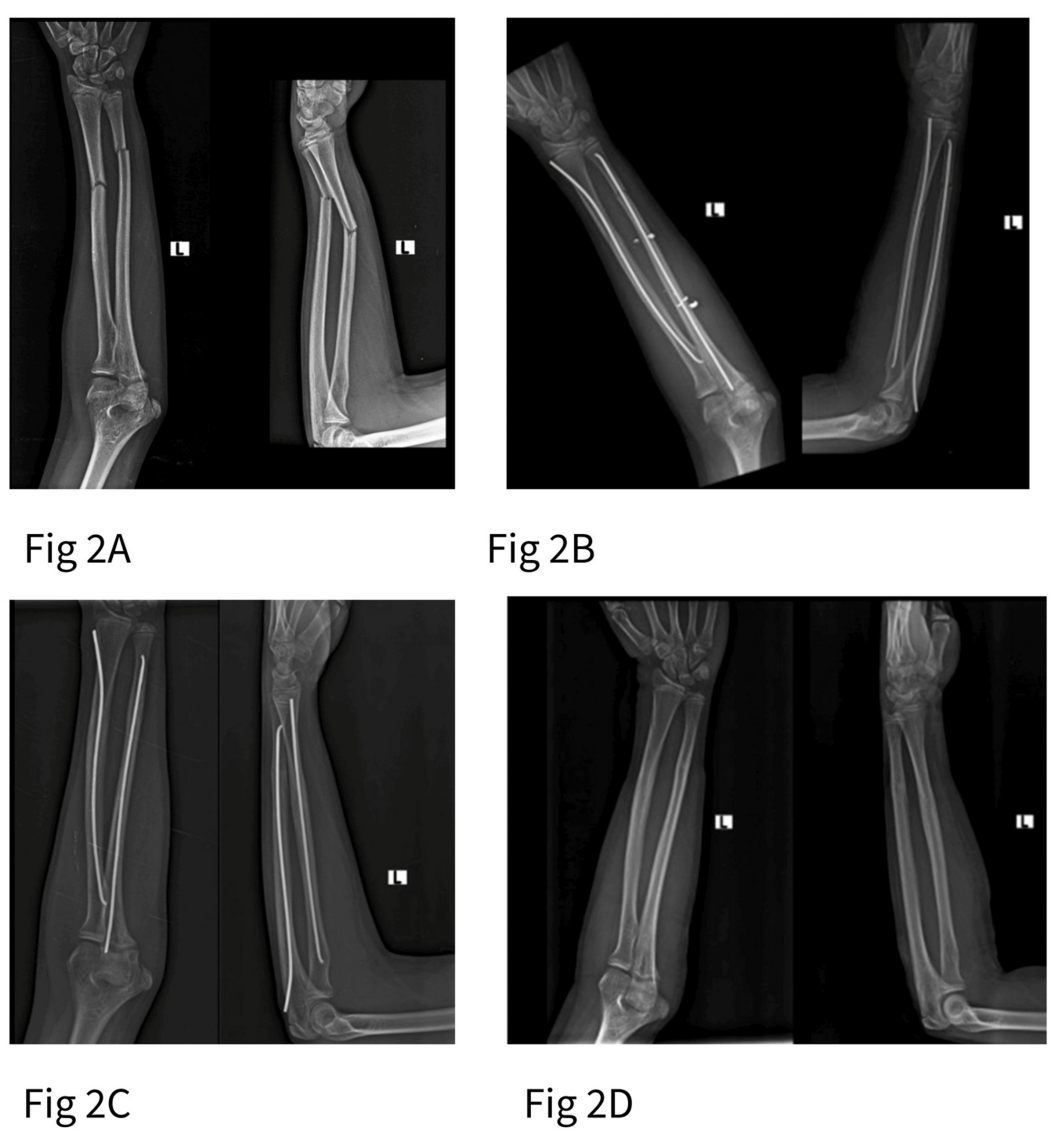
Plain radiographs representing antegrade titanium elastic nailing for left ulna fracture A: Pre-operative; B: Immediate post-operative; C: 6 months follow-up with complete union; D: post implant removal.

Retrograde nailing achieved faster unadjusted radiographic union (7.4 ± 0.6 weeks versus 8.6 ± 0.7 weeks; t=7.74; df=68; p<0.001). The retrograde group had more distal-third fractures, and distal fractures heal faster regardless of technique. After analysis of covariance (ANCOVA) adjustment for fracture location, the mean difference dropped to 0.6 weeks (95% CI 0.01-1.18; F=4.10; df=1,67; p=0.047), the case mix accounts for much of the raw difference. Although statistically significant, this is not a difference that changes management. All fractures united by 10 weeks in both groups. Representative radiological progression for each technique, from preoperative imaging through union and implant removal, is illustrated in Figures 1, 2.

Functional recovery was excellent and equivalent in both groups at six months (Table 2).

**Table 2:** Radiographic and functional outcomes Values are presented as mean ± SD or n (%). ^*^p<0.05 considered statistically significant; ^†^Adjusted for fracture location using ANCOVA (F-value and df reported). Continuous variables compared using independent t-test (t-value and df reported); Full ROM compared using Fisher’s exact test; DASH: Disabilities of the Arm, Shoulder and Hand; ROM: range of motion; df: degrees of freedom; SD: standard deviation.

Outcome measure	Antegrade (n=35)	Retrograde (n=35)	Test statistic	p-value
Radiographic union (weeks)	8.6 ± 0.7	7.4 ± 0.6	t = 7.74; df = 68	<0.001^*^
Adjusted union time (weeks)^†^	8.3 ± 0.5	7.7 ± 0.5	F = 4.10; df = 1,67	0.047^*^
DASH score at six months	4.2 ± 2.1	3.6 ± 1.8	t = 1.28; df = 68	0.198
Full ROM at six months	32 (91.4%)	33 (94.3%)	Fisher’s exact	0.642
Elbow flexion (°)	138.6 ± 4.2	140.2 ± 3.8	t = 1.77; df = 68	0.081
Elbow extension deficit (°)	2.8 ± 1.6	2.2 ± 1.4	t = 1.79; df = 68	0.078
Forearm pronation (°)	78.2 ± 4.1	79.5 ± 3.8	t = 1.38; df = 68	0.174
Forearm supination (°)	77.8 ± 4.5	79.1 ± 3.6	t = 1.34; df = 68	0.192
Time to implant removal (months)	4.8 ± 1.2	5.1 ± 1.0	t = 1.11; df = 68	0.268

Full ROM was achieved by 32 (91.4%) antegrade and 33 (94.3%) retrograde patients (Fisher’s exact; p=0.642). Mean DASH scores were 4.2 ± 2.1 versus 3.6 ± 1.8 (t=1.28; df=68; p=0.198). Elbow flexion was 138.6 ± 4.2° versus 140.2 ± 3.8° (t=1.77; df=68; p=0.081), and extension deficit was 2.8 ± 1.6° versus 2.2 ± 1.4° (t=1.79; df=68; p=0.078). Forearm pronation and supination were similarly equivalent between groups. No significant difference was found in any functional parameter. Preoperative ROM and DASH scores were not available, so the six-month values represent functional status at follow-up rather than change attributable to surgery.

Complications

Complication analysis revealed significantly fewer implant-related issues in the retrograde group (Table 3).

**Table 3:** Comparison of complications between the groups Values are presented as n (%). *p<0.05 considered statistically significant. Chi-squared test (χ²) used for cells with expected frequency ≥5; Fisher’s exact test used where expected frequency <5; χ²: chi-squared statistic; df: degrees of freedom.

Complication	Antegrade (n=35)	Retrograde (n=35)	Test statistic	p-value
Olecranon irritation	12 (34.3%)	0 (0%)	χ² = 14.48; df = 1	<0.001*
Nail back-out/migration	4 (11.4%)	2 (5.7%)	Fisher’s exact	0.394
Superficial infection	2 (5.7%)	1 (2.9%)	Fisher’s exact	0.556
Delayed union	1 (2.9%)	0 (0%)	Fisher’s exact	0.314
Nail proximity to physis	0 (0%)	2 (5.7%)	Fisher’s exact	0.152
Early implant removal	8 (22.9%)	0 (0%)	χ² = 9.03; df = 1	<0.001*
Total patients with complications	20 (57.1%)	5 (14.3%)	χ² = 14.00; df = 1	<0.001*
Complication-free rate	15 (42.9%)	30 (85.7%)	χ² = 14.00; df = 1	<0.001*

Olecranon irritation was the most common complication in the antegrade group: 12 (34.3%) patients and was entirely absent in the retrograde group (χ² = 14.48; df= 1; p<0.001). Early implant removal due to symptomatic hardware was required in eight (22.9%) patients in the antegrade group versus 0 (0%) in the retrograde group (χ² = 9.03; df=1; p<0.001). The overall complication-free rate significantly favored the retrograde technique: 30 (85.7%) versus 15 (42.9%) (χ² = 14.00; df=1; p<0.001). Bonferroni correction was applied across multiple comparisons; both olecranon irritation and early implant removal held up to the corrected threshold. Two younger patients in the retrograde group demonstrated nail proximity to the distal physis without early growth disturbance; however, long-term follow-up remains necessary.

## Discussion

Seventy children with closed ulnar diaphyseal fractures were treated with either antegrade or retrograde TEN over two years at our institution. The key findings were straightforward: retrograde nailing had fewer implant complications and comparable functional recovery. The union time difference, once case mix was accounted for, was too small to matter clinically.

Radiological union

Retrograde nailing united faster on raw numbers - 7.4 vs. 8.6 weeks - consistent with Thürig et al. [8], who reported biomechanical advantages in distal and midshaft fractures. The problem is that the retrograde group had more distal fractures to begin with (40.0% vs. 25.7%), and distal fractures heal faster regardless of how they are fixed. Once the fracture location was included as a covariate, the difference dropped to 0.6 weeks. Although statistically significant (F=4.10; p=0.047), this is not a difference that changes management. What the data do confirm is that all fractures united by ten weeks in both groups. Neither technique failed to achieve union. All functional parameters at six months were equivalent between groups. DASH scores were 4.2 vs. 3.6 (t=1.28; p=0.198), full ROM exceeded 90% in both cohorts, elbow flexion reached 138.6° vs. 140.2° (t=1.77; p=0.081), and extension deficits were minimal at 2.8° vs. 2.2° (t=1.79; p=0.078). These near-normal elbow values are expected - the fracture is diaphyseal and remote from the joint, so periarticular stiffness is uncommon. Jain et al. [13], Somisettty et al. [14], and Saseendar et al. [15] found similar results using TENS across different patient populations and technique variations. Nail entry point does not appear to drive functional recovery. Rehabilitation quality and fracture biology likely matter more. One important gap: preoperative scores were not collected, so we cannot quantify how much each group actually improved. The six-month values show where patients ended up, not how far they came.

Complication profiles

The complication picture was the clearest finding in this study. Eighty-six per cent (30/35) of retrograde patients had no complications at all. Only 43% (15/35) of the antegrade group could say the same. Olecranon irritation was the main culprit, affecting 12 (34.3%) antegrade patients and none in the retrograde group. This is not surprising. The olecranon has very little soft tissue over it, particularly in lean children, and even a small amount of nail protrusion can cause persistent irritation [10]. Johnson et al. [6] reported a nearly four-fold higher rate of symptomatic hardware with antegrade insertion, which fits our experience. To account for testing multiple complications simultaneously, Bonferroni correction was applied. Both olecranon irritation (χ² = 14.48; p<0.001) and early implant removal (χ² = 9.03; p<0.001) held up to the corrected threshold. These results favor retrograde entry for fractures where the technique is anatomically appropriate. The two young retrograde patients with physeal proximity are worth discussing separately. Both were under eight years old, an age group where the distal ulnar physis is still wide open and vulnerable. Neither showed changes at six months. That is encouraging but inconclusive. Madelung-type deformity after physeal arrest from distal ulnar instrumentation can take years to appear [16]. We do not yet know the full outcome for these two children. Long-term monitoring is advisable for any young patient where the retrograde entry point is close to the physis.

Surgical decision framework

Based on these findings, retrograde nailing appears to be the better option for midshaft and distal ulnar fractures in children aged 10 and above. The complication burden is lower, and the soft tissue anatomy at the distal entry site is more forgiving. For proximal fractures, antegrade nailing is technically more appropriate, a retrograde nail traversing the full medullary canal in a proximal fracture is mechanically unfavorable. In children under eight, particularly those with widely open physes, the distal entry carries a degree of uncertainty that justifies choosing antegrade instead. Nail sizing at approximately 80% of canal diameter and keeping tail protrusion to 5-10 mm apply to both approaches and reduce hardware irritation regardless of entry point [17].

Limitations

This study has real weaknesses. The most important thing is that it was not randomized. Surgeons chose the approach based on fracture location, which meant the retrograde group ended up with more distal fractures. Distal fractures heal faster. We tried to account for this statistically, but because the study was not randomized, some selection bias may have influenced the results. The union time advantage cannot be attributed to the technique with any confidence. Six months is not enough follow-up for the physeal question. Growth arrest after distal ulnar entry may not show up for two to five years. Our two youngest retrograde patients looked fine at six months - that does not mean they are fine. Both are being monitored. We also did not record preoperative DASH scores, which makes it impossible to measure how much patients actually improved. Finally, the cohort mixed both-bone forearm fractures with isolated ulnar fractures, and the study was conducted at a single center. These factors limit how broadly the findings can be applied.

## Conclusions

Both antegrade and retrograde titanium elastic nailing provide reliable union and excellent functional recovery in pediatric ulnar diaphyseal fractures. Retrograde nailing produced far fewer implant-related problems, mainly because it avoided the olecranon, a site with almost no soft tissue buffer in thin children. For midshaft and distal fractures in older children, retrograde nailing is the better choice. For proximal fractures and for children under eight with open physes, antegrade nailing is more appropriate. Prospective randomized trials with larger sample sizes and long-term follow-up to skeletal maturity are needed before the physeal question can be answered definitively.

## References

[1] Landin LA (1997). Epidemiology of children's fractures. J Pediatr Orthop B.

[2] Khosla S, Melton LJ 3rd, Dekutoski MB, Achenbach SJ, Oberg AL, Riggs BL (2003). Incidence of childhood distal forearm fractures over 30 years: a population-based study. JAMA.

[3] Lyons RA, Delahunty AM, Kraus D, Heaven M, McCabe M, Allen H, Nash P (1999). Children's fractures: a population based study. Inj Prev.

[4] Lascombes P, Prevot J, Ligier JN, Metaizeau JP, Poncelet T (1990). Elastic stable intramedullary nailing in forearm shaft fractures in children: 85 cases. J Pediatr Orthop.

[5] Flynn JM, Jones KJ, Garner MR, Goebel J (2010). Eleven years experience in the operative management of pediatric forearm fractures. J Pediatr Orthop.

[6] Johnson TR, Haus AJ, Shah KN, Bankole AI, Hogue GD (2020). Antegrade elastic intramedullary nailing insertion technique results in higher incidence of symptomatic implants in pediatric ulnar fractures. J Am Acad Orthop Surg Glob Res Rev.

[7] Nandyala SV, Shore BJ, Hogue GD (2021). Pearls and pitfalls of forearm nailing. J Pediatr Orthop Soc North Am.

[8] Thürig G, Raabe I, Maniglio M, Vial P, Tannast M, Gautier E (2022). Retrograde intramedullary nailing for bado types proximal pediatric ulna fractures: different surgical techniques. J Pediatr Perinatol Child Health.

[9] von Elm E, Altman DG, Egger M, Pocock SJ, Gøtzsche PC, Vandenbroucke JP (2007). The strengthening the reporting of observational studies in epidemiology (STROBE) statement: guidelines for reporting observational studies. Lancet.

[10] Martus JE, Preston RK, Schoenecker JG, Lovejoy SA, Green NE, Mencio GA (2013). Complications and outcomes of diaphyseal forearm fracture intramedullary nailing: a comparison of pediatric and adolescent age groups. J Pediatr Orthop.

[11] Papamerkouriou YM, Christodoulou M, Krallis P, Rajan R, Anastasopoulos J (2020). Retrograde fixation of the ulna in pediatric forearm fractures treated with elastic stable intramedullary nailing. Cureus.

[12] Hudak PL, Amadio PC, Bombardier C (1996). Development of an upper extremity outcome measure: the DASH (disabilities of the arm, shoulder and hand). Am J Ind Med.

[13] Jain S, Mohanachandran J, Mohan R (2023). Outcomes and complications of titanium elastic nailing for forearm bones fracture in children: our experience in a district general hospital in the United Kingdom. Acta Orthop Belg.

[14] Somisetty TK, Seenappa H, Kumar K V, Shanthappa AH, P A (2023). Evaluation of the functional outcome of both bone forearm fractures in the pediatric population with the titanium elastic nailing system in a tertiary care center. Cureus.

[15] Saseendar S, Kp UA, Latchoumibady K, Shanmugasundaram S (2024). Pediatric forearm fractures: investigating the functional outcomes of titanium elastic nailing for unstable both-bone fractures. J Orthop Case Rep.

[16] Meirizal Meirizal, Muhammad H, Rukmoyo T, Angriawan T (2023). Management of pediatric Madelung's deformity of the forearm due to physeal growth arrest of the distal ulna: a case report. Ann Med Surg (Lond).

[17] Kapoor V, Theruvil B, Edwards SE, Taylor GR, Clarke NM, Uglow MG (2005). Flexible intramedullary nailing of displaced diaphyseal forearm fractures in children. Injury.

